# Identifying Explainable Machine Learning Models and a Novel SFRP2^+^ Fibroblast Signature as Predictors for Precision Medicine in Ovarian Cancer

**DOI:** 10.3390/ijms242316942

**Published:** 2023-11-29

**Authors:** Ziyi Yang, Dandan Zhou, Jun Huang

**Affiliations:** School of Life Sciences, Zhengzhou University, Zhengzhou 450001, China

**Keywords:** ovarian cancer, machine learning, SFRP2, single-cell analysis, fibroblast, precision medicine

## Abstract

Ovarian cancer (OC) is a type of malignant tumor with a consistently high mortality rate. The diagnosis of early-stage OC and identification of functional subsets in the tumor microenvironment are essential to the development of patient management strategies. However, the development of robust models remains unsatisfactory. We aimed to utilize artificial intelligence and single-cell analysis to address this issue. Two independent datasets were screened from the Gene Expression Omnibus (GEO) database and processed to obtain overlapping differentially expressed genes (DEGs) in stage II–IV vs. stage I diseases. Three explainable machine learning algorithms were integrated to construct models that could determine the tumor stage and extract important characteristic genes as diagnostic biomarkers. Correlations between cancer-associated fibroblast (CAF) infiltration and characteristic gene expression were analyzed using TIMER2.0 and their relationship with survival rates was comprehensively explored via the Kaplan–Meier plotter (KM-plotter) online database. The specific expression of characteristic genes in fibroblast subsets was investigated through single-cell analysis. A novel fibroblast subset signature was explored to predict immune checkpoint inhibitor (ICI) response and oncogene mutation through Tumor Immune Dysfunction and Exclusion (TIDE) and artificial neural network algorithms, respectively. We found that Support Vector Machine–Shapley Additive Explanations (SVM-SHAP), Extreme Gradient Boosting (XGBoost), and Random Forest (RF) successfully diagnosed early-stage OC (stage I). The area under the receiver operating characteristic curves (AUCs) of these models exceeded 0.990. Their overlapping characteristic gene, secreted frizzled-related protein 2 (SFRP2), was a risk factor that affected the overall survival of OC patients with stage II–IV disease (log-rank test: *p* < 0.01) and was specifically expressed in a fibroblast subset. Finally, the SFRP2^+^ fibroblast signature served as a novel predictor in evaluating ICI response and exploring pan-cancer tumor protein P53 (TP53) mutation (AUC = 0.853, 95% confidence interval [CI]: 0.829–0.877). In conclusion, the models based on SVM-SHAP, XGBoost, and RF enabled the early detection of OC for clinical decision making, and SFRP2^+^ fibroblast signature used in diagnostic models can inform OC treatment selection and offer pan-cancer TP53 mutation detection.

## 1. Introduction

Ovarian cancer (OC), cervical cancer, and endometrial cancer are commonly diagnosed diseases that affect the female reproductive organs [1,2,3]. Advanced-stage OC is estimated to have the highest mortality rate among gynecological cancers [4,5,6]. Approximately 90–95% of OC takes the form of primary tumors originating from epithelial cells, while the remaining 5–10% comprises primary cancers from other cell subtypes [3,6,7]. OC can be staged based on the extent of the spread [8,9]. In stage I, the tumor is confined to either the ovaries or fallopian tubes [8]; the 5-year survival rate of patients in this stage is approximately 89% [9,10]. In stage II, the tumor involves one or both ovaries and has spread within the pelvis. The 5-year survival rate of stage II OC patients is approximately 70%. Stage III involves the infiltration of one or both ovaries, with extra pelvic peritoneal metastasis or retroperitoneal lymph node metastasis confirmed cytologically or histologically. The 5-year survival rates for OC patients with stage III is approximately 36%. Stage IV signifies the presence of distant metastasis beyond the abdominal cavity. This includes positive pleural effusion cytology, metastasis to the parenchyma of the liver or spleen, metastasis to extra-abdominal organs (such as inguinal lymph nodes and lymph nodes outside the abdominal cavity), as well as transmural invasion of the intestinal tract. The 5-year survival rate of patients in this stage is low, at around 17%.

The treatment for OC patients generally includes surgical intervention, along with options for radiotherapy or chemotherapy [11,12]. Early-stage OC patients are candidates for comprehensive staging surgery, which is used to accurately determine the stage of the disease [11,13]; tumor cytoreductive surgery is suitable for moderate- to advanced-stage patients with extraovarian metastases [14]. Paclitaxel combined with carboplatin is the drug of choice for first-line neoadjuvant chemotherapy, and the combination chemotherapy of taxane/platinum or doxorubicin liposome/carboplatin is a common postoperative adjuvant chemotherapy [15,16]. Bevacizumab is a prominent drug in anti-angiogenic therapy. It is administered in combination with chemotherapy and is suitable for both first-line treatment and the treatment of relapsed OC cases [17,18,19]. Currently, olaparib, niraparib, and fluzoparib are poly ADP-ribose polymerase (PARP) inhibitors that have received marketing approval. These drugs have demonstratable effectiveness in the maintenance treatment of OC [20,21,22]. Due to the absence of noticeable symptoms in its early stages, the detection of OC in this stage is highly challenging [11,23]. The majority of OC cases are diagnosed at a late stage, which greatly reduces the effectiveness of curative treatments [5]. Consequently, the overall prognosis for OC is generally low [24,25]. The effectiveness of first-line chemotherapy is greater, but a significant proportion of patients will relapse, with a very short average recurrence time [26,27,28]. Therefore, the development of clinical biomarkers and robust predictive models that can accurately diagnose early-stage OC is of utmost urgency.

There has been a significant surge in the amount and complexity of data generated from individuals and biological experiments as a result of numerous research efforts and breakthroughs in the biomedical field in recent years. This presents both opportunities and challenges for precision medicine [29,30,31,32]. The exponential expansion of existing biomedical data has surpassed the capacity to utilize traditional methods to extract meaningful insights and conduct in-depth studies on irradicable diseases. This necessitates the development of novel approaches that can assist researchers in effectively handling and interpreting vast and intricate datasets [29,33]. There has been a growing trend in utilizing machine learning methods to meet specific requirements. Machine learning, an artificial intelligence technology, enables computers to learn from the large scale of data features, identify specific patterns, and automatically uncover correlations and distinctions among different objects. It facilitates the rapid construction of diagnostic and prognostic models and often demonstrates good predictive performance [31,34]. For instance, Liu created SVM models to detect early OC and prostate cancer [35]. Klein et al. applied five machine learning algorithms to jointly complete the subtype distinction of EOC tissue [36]. Gevaert et al. reported Least Squares Support Vector Machines (LS-SVMs) in the prediction of OC in advanced stages [37]. These studies constructed models with good performance in OC detection, but did not provide explanations regarding their underlying mechanisms in terms of the rank of feature importance. Explainable machine learning methods, such as Random Forest (RF) and Extreme Gradient Boosting (XGBoost), offer promising solutions to address this issue. RF is an algorithm that utilizes multiple trees during the training process. The fundamental component of RF is the decision tree, where each classification tree is constructed from a random subset of the input data, consisting of input and output variables [38,39]. XGBoost is another machine learning algorithm widely used in classification and regression tasks, which uses the principle of the gradient descent algorithm to boost weak learners, based on gradient boosting trees, and adopts an additive strategy [40,41,42]. Shapley Additive Explanations (SHAP), a “model explanation” package developed in Python, allows us to analyze each feature’s contribution to the model’s decision-making process [43,44,45]. Combining Support Vector Machine (SVM) and SHAP facilitates feature selection [46], making SVM models easier to follow, especially for applications that require interpretability. Indeed, several studies utilized the explainable machine learning models for various aspects of OC diagnosis and have achieved significant progress in this regard [47,48,49]. However, to our knowledge, the joint application of SVM-SHAP, XGBoost, and RF in the efficient diagnosis of OC in the early stage (stage I) remains unsatisfactory. It is worth conducting research on these interpretable machine learning models to explore their ability with excellent diagnostic performance in early-stage OC diagnosis and to identify characteristic genes as prognostic factors.

The tumor microenvironment (TME) plays an important role in a tumor’s development, which provides potential therapeutic targets for treating cancer [50,51]. Cancer-associated fibroblasts (CAFs) form protective structures that can promote the progression of matrix formation and unfavorable TME to support tumor growth [52]. CAFs can release a variety of cytokines that affect different signaling pathways to promote the proliferation of surrounding tumor cells within the TME [51]. CAFs are key stromal components of the metastatic niche that can secrete pro-metastatic cytokines to facilitate tumor dissemination, promoting cancer cell peritoneal metastasis in ovarian cancer patients [53]. CAFs also interact with immune components to mediate the formation of a suppressive antitumor microenvironment [54]; several CAF subtypes may promote chemoresistance by maintaining cancer cell stemness [55]. In addition, different subtypes of CAFs exhibit specific functions in tumor pathogenesis [56]. However, the co-enrichment between the expression level of feature genes yielded by interpretable machine learning methods and CAFs infiltrates abundance in OC patients, as well as their cellular location in the TME, are far from being investigated.

In this study, our primary aim was to construct robust explainable machine learning models that can effectively identify whether the tumor is confined to the ovaries or fallopian tubes. Then, we investigated the performance of intersecting important feature genes derived from explainable machine learning algorithms in relation to determining OC prognosis and its localization in fibroblast subpopulations. Finally, we evaluated a unique fibroblast signature characterized by a specific gene expression pattern as candidate indicators for diagnosing the sensitivity of OC patients to ICI therapy and the presence of tumor protein p53 (*TP53*) mutations in pan-cancer samples.

## 2. Results

### 2.1. Acquisition of Differentially Expressed Genes (DEGs)

**Figure 1:**
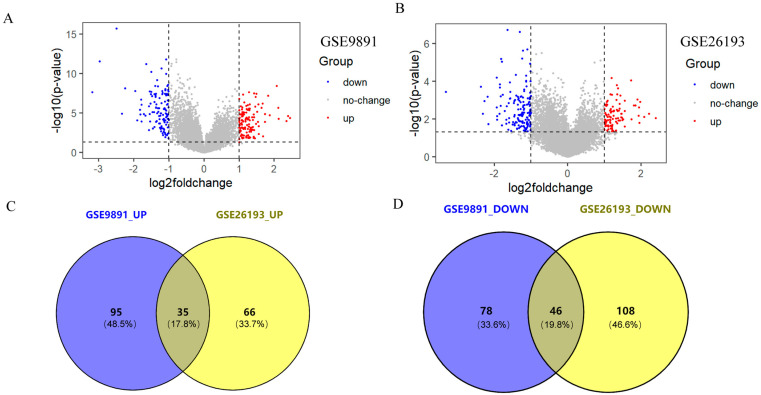
Distribution of DEGs in the GEO datasets. (**A**) Volcanic map showing the distribution of dysregulated genes in dataset GSE9891. Blue scatter points are downregulated genes, red scatter points are upregulated genes, and gray scatter points are non-characteristic genes. (**B**) A volcanic map showing the distribution of dysregulated genes in dataset GSE26193. Blue scatter points are downregulated genes, red scatter points are upregulated genes, and gray scatter points are non-characteristic genes. (**C**) Venn diagram showing the number of upregulated genes in the datasets GSE9891 and GSE26193; the intersection part is the total number of upregulated genes in the two datasets, the remaining blue part is the number of upregulated genes unique to GSE9891, and the remaining yellow part is the number of upregulated genes unique to GSE26193. (**D**) Venn diagram showing the number of downregulated genes shared between datasets GSE9891 and GSE26193; the intersection part is the number of downregulated genes shared between the two datasets, the remaining blue part is the number of downregulated genes unique to GSE9891, and the remaining yellow part is the number of downregulated genes unique to GSE26193.

There were 254 DEGs in GSE9891, including 130 upregulated genes and 124 downregulated genes in stage II-IV patients compared to early-stage (stage I) patients (Figure 1A). Meanwhile, 255 DEGs were identified in GSE26193, including 101 upregulated genes and 154 downregulated genes (Figure 1B). Then, Veen analysis was utilized to identify the common upregulated and downregulated genes in both GSE9891 and GSE26193, resulting in a total of 81 overlapping DEGs, including 35 upregulated genes and 46 downregulated genes (Figure 1C,D).

### 2.2. Functional Enrichment Analysis

**Figure 2:**
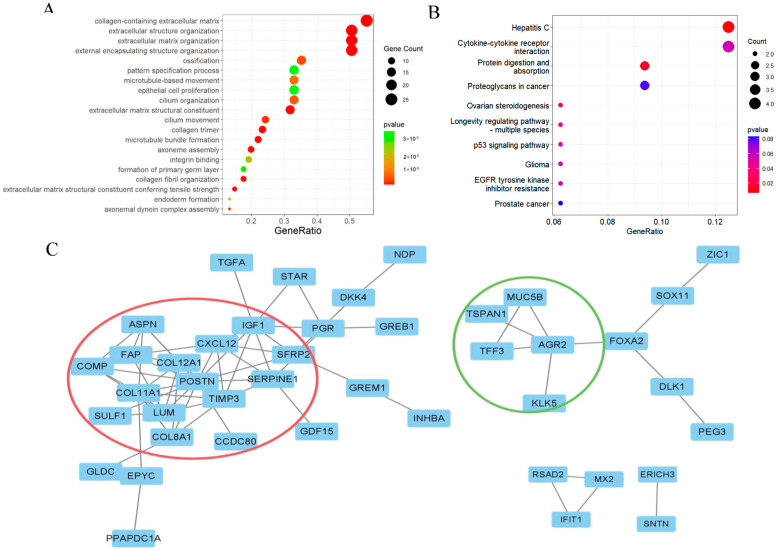
Results of function enrichment analysis of overlapping DEGs. (**A**) Results of GO analysis of DEGs. (**B**) Results of KEGG analysis of DEGs. (**C**) PPI network analysis and visualization results of overlapping DEGs using Cytoscape.

Based on the results of the Gene Ontology (GO) analysis, the following biological functions were significantly enriched: collagen-containing extracellular matrix, extracellular structure organization, extracellular matrix organization, and external encapsulating structure organization (Figure 2A). The Kyoto Encyclopedia of Genes and Genomes (KEGG) analysis also revealed significant enrichment of pathways such as Hepatitis C, protein digestion and absorption (Figure 2B). Eighty-one differentially expressed genes were analyzed using the Search Tool for the Retrieval of Interacting Genes/Proteins (STRING) website, revealing interactions between their encoding proteins. The core Protein–Protein Interaction (PPI) network was constructed in STRING (Kmeans cluster) and visualized using Cytoscape, consisting of 42 nodes and 69 edges (Figure 2C).

### 2.3. Screening 12 DEGs as Diagnostic Markers for Detecting Early-Stage OC Patients

**Figure 3:**
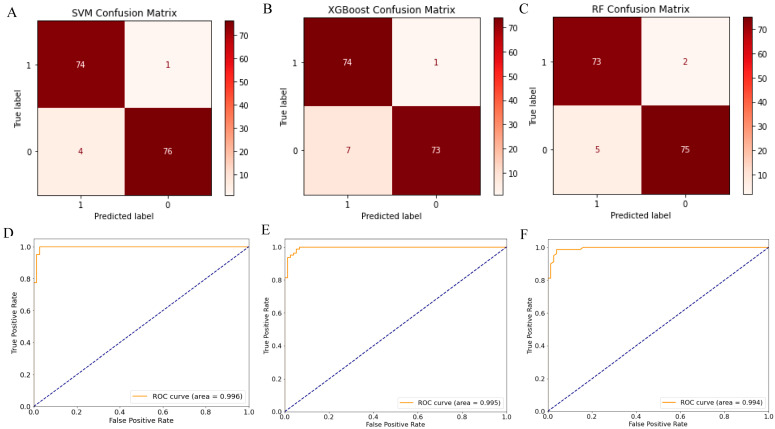
Performance analysis results including confusion matrix and receiver operating characteristic (ROC) curve of machine learning model. The red box in the confusion matrix is the number of samples predicted accurately, and the off-white box is the number of samples predicted incorrectly. The receiver operating characteristic (ROC) curve shows the area under the maximum curve (AUC), which can be used as a basis to judge the performance of a machine learning model. (**A**) Confusion matrix of Support Vector Machine (SVM) algorithm. (**B**) Confusion matrix of Extreme Gradient Lift (XGBoost) algorithm. (**C**) Confusion matrix of Random Forest (RF) algorithm. (**D**) ROC curve of SVM. (**E**) ROC curve of XGBoost. (**F**) ROC curve of RF.

**Figure 4:**
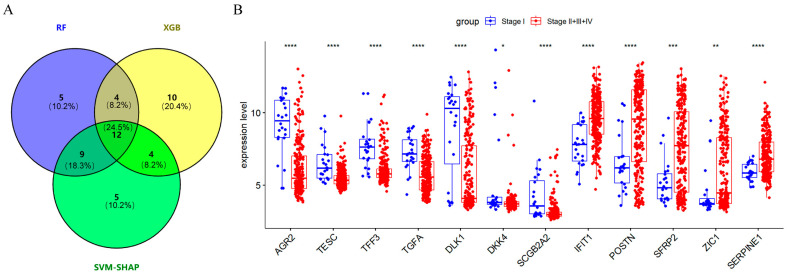
Number of feature genes screened using different machine learning algorithms and the difference in these genes’ expression level. The number of feature genes shared by the three machine learning model algorithms is shown in (**A**). Blue represents the number of feature genes screened by the Random Forest (RF) algorithm, yellow represents the number of feature genes screened by the Extreme Gradient Boost (XGBoost) algorithm, and green represents the number of feature genes screened by the Support Vector Machine (SVM) algorithm. (**B**) Comparison of expression levels of 12 characteristic genes in pathological stage I and other stages in GSE9891. * *p* < 0.05, ** *p* < 0.01, *** *p* < 0.001 and **** *p* < 0.0001.

**Figure 5:**
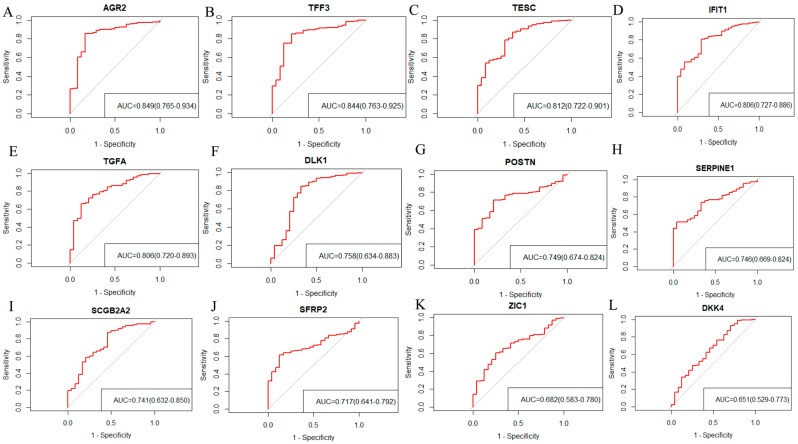
ROC curves of 12 characteristic genes. (**A**–**L**) describe the ROC curves and AUC values of 12 characteristic genes.

The confusion matrices of each machine learning model were shown in Figure 3A–C. Alongside the area under curve (AUC) values with a 95% confidence interval (CI) of SVM-SHAP, XGBoost, and RF, these were used to predict whether the tumors of stage I OC patients were 0.996 (95% CI: 0.992–1.000), 0.995 (95% CI: 0.990–1.000) and 0.994 (95% CI: 0.985–1.000), respectively (Figure 3D–F). The clinical utility index (CUI) of the SVM model was 0.936–0.938, the XGBoost model was 0.900–0.901, and the RF model was 0.911–0.913, respectively. The CUI values of these models were greater than 0.81, indicating that these models may have excellent utility in clinical practice for the diagnosis of stage I ovarian cancer. Furthermore, the top 30 important feature genes were screened, among which *AGR2*, *TESC*, *TFF3*, *TGFA*, *DLK1*, *DKK4, SCGB2A2*, *IFIT1*, *POSTN*, *SFRP2*, *ZIC1*, and *SERPINE1* were the overlapping important genes in three algorithms (Figure 4A), including seven downregulated genes (*AGR2*, *TESC*, *TFF3*, *TGFA*, *DLK1*, *DKK4*, and *SCGB2A2*) and five upregulated genes (*IFIT1*, *POSTN*, *SFRP2*, *ZIC1*, and *SERPINE1*) (Figure 4B). This study evaluated the diagnostic value of 12 feature genes as biomarkers for determining whether the tumor of ovarian cancer patients will metastasize outside the ovary, as shown in Figure 5. Genes such as *AGR2*, *TFF3*, *TESC*, *IFIT1*, *TGFA*, *DLK1*, *POSTN*, *SERPINE1*, *SCGB2A2*, and *SFRP2* with AUC values between 0.7 and 0.9 can independently predict whether a tumor is localized in the ovaries or in the fallopian tubes (Figure 5).

### 2.4. SFRP2 and SERPINE1 Were Intricately Linked to Cancer-Associated Fibroblasts and Associated with the Overall Survival of Patients in Moderate to Advanced-Stage OC

**Figure 6:**
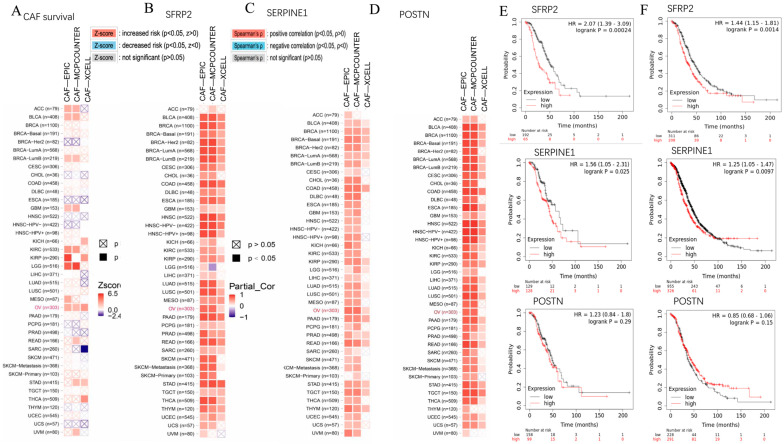
Co-enrichment of three characteristic genes with CAFs and Kaplan-Meier survival analysis. (**A**) Heatmaps demonstrate the correlation between enrichment level of CAFs and the survival risk of tumor patients through pan-cancer analysis. The font for OC is highlighted in red, while the font for other types of tumors is displayed in black. (**B**–**D**) show the correlation between the enrichment levels of CAFs and expression levels of *SFRP2*, *SERPINE1*, and *POSTN*, respectively, via pan-cancer analysis. (**E**,**F**) represent the survival status of advanced OC patients with differential expression levels of genes *SFRP2*, *SERPINE1*, and *POSTN*, respectively, in the GSE9891 dataset and in the dataset encompassing all ovarian cancer patients.

Through pan-cancer analysis, we discovered that the levels of CAF infiltrates’ abundances were risk factors that affected the overall survival rates of OC patients, as indicated by the three aforementioned deconvolution methods: EPIC, MCPcounter, and xCell (Figure 6A). Among the 12 featured genes (*AGR2*, *TESC*, *TFF3*, *TGFA*, *DLK1*, *DKK4*, *SCGB2A2*, *IFIT1*, *POSTN*, *SFRP2*, *ZIC1*, and *SERPINE1*), *SFRP2* and *SERPINE1* showed a positive association with the enrichment level of CAFs, as generated via three immune deconvolution methods (Figure 6B,C). *POSTN* showed a positive association with CAFs, as generated via two immune deconvolution methods (Figure 6D). The results of the Kaplan–Meier plots indicate that the higher expression levels of both *SFRP2* and *SERPINE1* are associated with lower survival rates in the GSE9891 individual dataset (stage: II + III + IV) and the entire OC datasets (stage: II + III + IV), respectively (Figure 6E,F). Additionally, the log-rank test comparing higher and lower levels of the *SFRP2* groups demonstrated that the *p*-values were less than 0.01, indicating statistical significance. However, the expression levels of *POSTN* in OC patients did not show a significant correlation with survival rates.

### 2.5. SFRP2 Might Define a Distinct CAF Subpopulation

**Figure 7:**
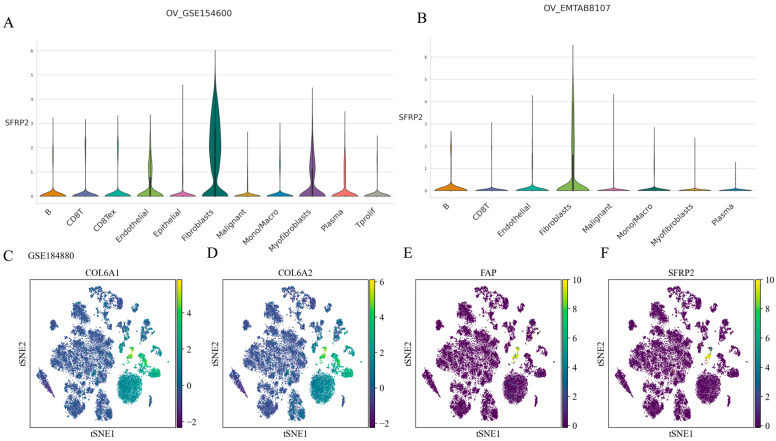
Identification of a novel CAF subset by Single-cell RNA sequencing analysis in OC. (**A**,**B**) Violin plot showing the expression levels of SFRP2 in each cell type in GSE154600 and EMTAB8107, respectively. (**C**,**D**) t-SNE plot showing cell clusters colored by *COL6A1* and *COL6A2*. (**E**,**F**) t-SNE plot showing cell clusters colored by *FAP* and *SFRP2*.

*SFRP2* was highly expressed in the fibroblast/myofibroblast subset in two independent datasets: GSE154600 (Figure 7A) and EMTAB8107 (Figure 7B), respectively. Furthermore, the violin plot demonstrates the distinct expression levels of SFRP2 in fibroblasts, suggesting the presence of a distinct subgroup. Consistently, single-cell RNA-seq analysis of nonmalignant tumor tissues and HGOSC tissues was utilized to investigate the distribution of *SFRP2* in TME and a novel subset with the highest enrichment of *SFRP2* and CAF markers (*COL6A1*, *COL6A2*, *FAP*) was identified (Figure 7C–F). These findings suggested the existence of a distinct CAF subgroup, which we named “SFRP2^+^ fibroblast”.

### 2.6. Contribution of SFRP2^+^ Fibroblast Signature in Predicting ICI Response and Detecting Pan-Cancer TP53 Mutation

**Figure 8:**
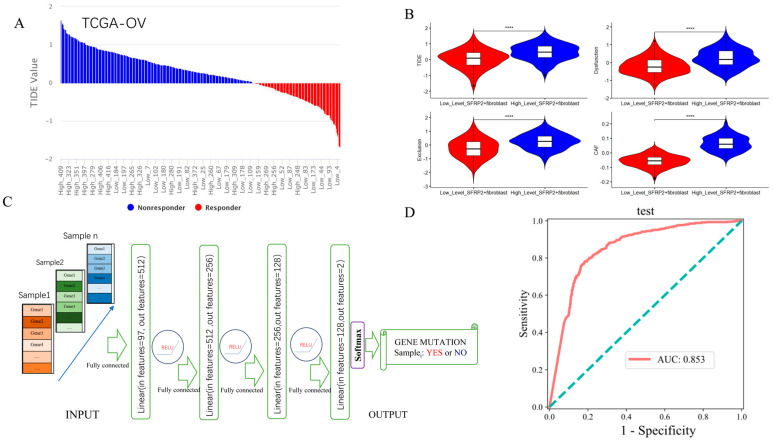
Top 100 specific genes from SFRP2^+^ fibroblast for predicting immune checkpoint inhibitor (ICI) responses and oncogene mutations. (**A**) Distribution of high-level SFRP2^+^ fibroblast and low-level SFRP2^+^ fibroblast among ICI responders and non-responders. (**B**) Violin plot showing differences in TIDE, Dysfunction, Exclusion, and CAF scores between the high-level SFRP2^+^ fibroblast and low-level SFRP2^+^ fibroblast groups within the TCGA−OV dataset. (**C**) Architecture diagram of the artificial neural network. (**D**) ROC curve of the performance of the model in testing sets for predicting *TP53* mutation in pan-cancer. **** *p* < 0.0001.

As shown in Figure 8A, OC patients from The Cancer Genome Atlas (TCGA-OV) with higher enrichment levels of SFRP2^+^ fibroblast mainly appeared in immune checkpoint inhibitor (ICI) non-responders, whereas the lower enrichment level of SFRP2^+^ fibroblast mainly appeared in ICI responders (Figure 8A). Significant differences in Tumor Immune Dysfunction and Exclusion (TIDE) scores, Dysfunction scores, Exclusion scores, and CAF scores between the two distinct groups were also observed (Figure 8B). The gene set composed of the top 100 specific genes of SFRP2^+^ fibroblast was explored in the Tumor Immune Single Cell Hub 2 (TISCH2), a single-cell data source, and was consistently found to be highly enriched in the fibroblast subset across various tumor types, suggesting their utility in pan-cancer analyses. An artificial neural network model based on the SFRP2^+^ fibroblast signature was established, and the main architecture of this model was presented in Figure 8C. This model demonstrated excellent performance in the test dataset, with an AUC value exceeding 0.85 (95% CI: 0.829–0.877) to distinguish between patients who bear the *TP53* mutation and those who do not (Figure 8D).

## 3. Discussion

Due to its inherent drug resistance and a high propensity for recurrence, the clinical outcomes of patients receiving therapies have fallen short of our expectations [12]. The 5-year survival rate remains stagnant at about 40%~50% [57,58]. The early diagnosis of OC can effectively improve survival rates, but only about 15% of OC patients are diagnosed in the early or local stages [59]. Thus, the construction of robust machine learning models for early diagnosis is a promising strategy for improving OC patients’ survival. To obtain input data suitable for machine learning, we primarily identified common DEGs that were upregulated and downregulated in OC patients in stages II–IV compared to those in the early stage in two independent GEO datasets. Through GO and KEGG pathway analysis, we found that common DEGs are related to tumor-associated signaling pathways and can regulate tumor progression [60]. In line with this, using these common DEGs as input, RF, SVM-SHAP, and XGBoost can distinguish stage I OC patients from patients in later stages. Compared with the general constructure of previous machine learning models, we used the Borderline Synthetic Minority Oversampling (BorderlineSMOTE) algorithm to uniformly optimize the data [61]. The importance of these three models using balanced data in predicting tumor stage was suggested by their high AUC values. Our results show that the machine learning models established with optimized balanced data performed better than those using unbalanced data.

The important feature genes employed in training outperformed models might also serve as diagnostic biomarkers themselves. The top 30 important features of each model were selected and intersected to obtain overlapping genes. One of them, *SFRP2*, is a member of the secreted frizzled-related protein (SFRP) family and a typical regulatory protein of the WNT pathway [62]; it has been reported as a co-factor for ten-eleven translocation 1 (TET1), contributing to the inhibition of tumor metastasis [63]. The stemness index (mRNAsi) has been quantified based on mRNA expression levels and *SFRP2* was one of genes associated with OC prognosis [57]. Furthermore, our studies revealed that *SFRP2* is a potential biomarker for the diagnosis of early-stage ovarian cancer with considerable AUC value and is associated with poor survival rates of OC patients in stages II-IV. Several studies reported that SFRP2 serves as a biomarker for breast cancer, in addition to various other cancers [64,65,66,67]. Our study underwent cross-validation with other studies highlighting the role of *SFRP2* in cancers and may lead to more significant findings. Thus, *SFRP2* may be an important gene with promising research prospects in pan-cancer.

The precise mechanism for obtaining the phenotype of CAF in cancers remains unclear. One of the principle underlying mechanisms may be the upregulation of SOX2, which could be inhibited by protein kinase Cζ (PKCζ) [68]. PKCζ deficiency can enable the upregulation of the WNT regulatory factor SFRP2, as SOX2 directly binds to the SFRP2 promoter. The inactivation of SFRP2 in CAFs impairs the induction of cancer cell migration and invasion and weakens the tumorigenicity of cancer cells in vivo [68]. This study highlights the potential role of SFRP2 in tumor pathogenesis. However, there are no reports detailing the cell subgroup localization of *SFRP2* in the TME of OC. To address this issue, we performed single-cell analysis and found that *SFRP2* is predominantly expressed in a unique subset of CAF in single-cell RNA-seq data from OC patients with nonmalignant tumor tissues and HGOSC tissues. Its functions are in need of further investigation.

Rapid progress is being made in the provision of valuable solutions, including diagnostic models based on extracellular matrix risk scores, autophagy-related signatures, and immune subtypes in the TME [69,70,71]. These models, which use transcriptome information, have clear feasibility with regard to screening an immunotherapy-advantage subgroup. As part of our research, the TIDE algorithm was employed to investigate the differences in immunotherapy response between OC patients with a higher and lower ssGSEA score for SFRP2^+^ fibroblast. We demonstrated that a significant majority of patients with high enrichment score of SFRP2^+^ fibroblast exhibited elevated TIDE scores, indicating that a high enrichment level of SFRP2^+^ fibroblast is correlated with a poor response to immunotherapy. The effectiveness of ICIs in ovarian cancer is limited; however, these findings based on the SFRP2^+^ fibroblast signature provide new insights for the assessment of immunotherapy and offer guidance on whether OC patients are likely to benefit from such treatments. As an interesting research direction in precision oncology, the detection of oncogene status in pan-cancer samples holds promise for advancing the clinical application of targeted therapy [72,73,74]. For some cases, knowing information about the oncogene status may have a significant impact on treatment options, facilitate appropriate clinical decision making, and guide the identification of suitable candidates for clinical trials. In this study, a deep artificial neural network-based predictive model was constructed by incorporating the SFRP2^+^ fibroblast signature to detect *TP53* mutation. Using expression data from less than 100 specific genes of SFRP2^+^ fibroblast as input, the model can effectively identify whether a patient harbors a *TP53* mutation, indicating the significance of tumor-associated fibroblast signature for deep learning and precision oncology. The pan-cancer analysis used in our model only included patients with *TP53* mutation levels higher than 0.3, and whether setting an even higher *TP53* mutation rate would be better to detect *TP53* mutations has yet to be determined. However, this artificial neural network model could not directly provide information regarding the correlation between levels of *SFRP2* expression and *TP53* mutations. Further exploration of their correlation, such as correlation coefficients, may also facilitate the development of novel biomarkers.

In conclusion, our study provided computer-aided diagnostic models with good performance for OC patient management. We believe that integrating the traditional and artificial intelligence-based predictive models constructed in our study can further benefit cancer patients.

This study had some limitations. It is important to note that expression of SFRP2 and the presence of SFRP2^+^ fibroblast in OC tumor tissues are still in need of further experimental validation. Evaluating artificial-intelligence-based predictive models in independent cohorts is necessary for their clinical applications in the future.

## 4. Materials and Methods

### 4.1. Data Collection and Processing

The GEO database on the National Center for Biotechnology Information’s (NCBI’s) website was accessed using the keyword “Ovarian Cancer” to retrieve microarray datasets, including GSE9891 and GSE26193. The information page of each dataset was then accessed, and the corresponding expression matrix and GPL platform were downloaded. Clinical sample information with varying disease conditions was contained in each dataset, while the GPL platform contained gene probes that corresponded to the expression matrix. Using the “GEOquery” R package, the expression matrix of each microarray dataset was preliminarily processed. The total number of samples and staging of each dataset were then organized and listed. GSE9891 and GSE26193 were selected for in-depth research after being screened for their large total number of samples and relatively uniform sample staging.

### 4.2. Screening and Analysis of Overlapping DEGs

This study utilized the “LIMMA” R package to analyze the datasets of GSE9891 and GSE26193, in order to identify DEGs in stage II-IV diseases vs. stage I diseases, and generated a volcano plot to visualize the differences. Upregulated differentially expressed genes with statistical significance were those with *p* < 0.05 and fold change (FC) ≥ 2. Downregulated differentially expressed genes with statistical significance were those with *p* < 0.05 and FC ≤ 0.5. The intersection of upregulated and downregulated genes in GSE9891 and GSE26193 was observed to obtain the common differentially expressed genes. The expression data of these common genes in GSE9891 were collected, and each sample was matched with its pathological stage. The final output was a gene expression matrix with a gene symbol.

### 4.3. Functional Analysis of Overlapping DEGs

After screening the DEGs, the “clusterProfiler” R package was utilized to conduct GO and KEGG pathway enrichment analysis on the differentially expressed genes. The purpose of this was to clarify the functions of the selected differentially expressed genes and the main biological processes they participate in. The data type of DEGs was converted from “SYMBOL” to “ENTREZID”, ignoring cases in which the gene names and IDs did not match, and the remaining genes were used for GO and KEGG pathway enrichment analysis. A threshold of *p* < 0.05 was considered statistically significant. This study also used the STRING website to analyze core protein–protein interactions (PPIs) among the overlapping DEGs. The Cytoscape plugin (cytohHubba) was used to visualize the PPI network obtained from the STRING website.

### 4.4. Explainable Machine Learning

Three explainable machine learning models including SVM-SHAP, XGBoost, and RF were employed in this study. These machine learning models, which used expression levels of common DEGs as input, were established to stage tumors and to identify important features as biomarkers. To address the data imbalance, the BorderlineSMOTE algorithm was used to oversample the data and then machine learning was performed. The Random Forest algorithm was run using the “RandomForest Classifier” function in the sklearn library in Python, and the 30 most important features were selected using the feature_importances_function. The SVM algorithm was also run using the “svm” function in the sklearn library, and the top 30 important feature genes were selected using the SHAP package. The XGBoost model was operated using the “XGB Classifier” package and the 30 most important features were selected using the feature_importances_ function. The performance of the machine learning models was comprehensively evaluated based on the confusion matrices, accuracy (ACC), recall (REC), F1 score, and the area under the curve (AUC) of the highest ROC curve. Overlapping important features were further analyzed and studied using the above three machine learning models. To further determine whether the selected genes could serve as biomarkers for the staging of ovarian cancer tumors, ROC curves of overlapping genes were evaluated using the GSE9891 dataset and their importance was assessed based on an AUC with 95% CI. The CUIs (+CUI, −CUI) of the three explainable machine learning models were also calculated [75].

### 4.5. Pan-Cancer Bioinformatics Analysis Using the TIMER2 Website

The pan-cancer analysis of gene expression and its correlation with CAF infiltrates’ abundances, as well as the correlation with clinical outcome, was conducted using the interactive webtool TIMER2 (http://timer.cistrome.org/, accessed on 27 November 2022). EPIC, MCPcounter, and xCell algorithms provided by TIMER2 were utilized to calculate the CAF infiltrates’ abundances. Original data were downloaded from TIMER2 and further visualized using R packages.

### 4.6. Survival Analysis

The Kaplan–Meier plotter website was utilized to plot the survival curves of the overlapping genes in both the individual dataset GSE9891 (stage: II + III + IV) and the entire ovarian cancer datasets (stage: II + III + IV). Hazard ratios (HRs) with corresponding 95% confidence intervals and log-rank *p*-values were calculated. A log-rank *p*-value less than 0.05 was considered statistically significant, indicating a difference in survival between patients classified into high and low expression groups based on overlapping genes.

### 4.7. Single-Cell RNA Sequencing Analysis

Data processing and visualization were performed using the Scanpy toolkit in Python for the GSE184880 dataset. The general steps involved selecting cells and genes, retaining cells with a minimum of 400 genes, keeping genes that appeared in at least 5 cells, filtering out cells with a high level of mitochondrial genes, and performing data standardization. Cell subpopulation clustering was conducted, and the top 100 genes in each subpopulation were identified.

The expression level of SFRP2 in different cell subpopulations in OC was also explored via individual gene exploration functions provided by an scRNA-seq database named Tumor Immune Single-cell Hub 2 (TISCH2).

### 4.8. Single-Sample Gene Set Enrichment Analysis (ssGSEA)

The enrichment levels of a given gene set in a single sample were assessed primarily using a specialized GSEA method, ssGSEA, provided by the “GSVA” R package. It provides scores for cell subpopulations, functional pathways, and other gene sets within a single tumor sample [76].

### 4.9. Tumor Immune Dysfunction and Exclusion (TIDE) Analysis

TIDE (http://tide.dfci.harvard.edu/login/, accessed on 10 February 2023) was employed in TCGA-OV patients to predict ICI responses, including the assessment of TIDE, Dysfunction, Exclusion, and CAF scores. Patients with TIDE information were then split into a group with higher-level SFRP2^+^ fibroblast and a group with lower-level SFRP2^+^ fibroblast based on the median ssGSEA scores of a gene set composed of the top 100 specific genes in SFRP2^+^ fibroblast. The results were further visualized using the “ggpubr” R package.

### 4.10. Identification of Pan-Cancer TP53 Mutation by Artificial Neural Network

Pan-cancer mutation burden data, pan-cancer mutation information data, pan-cancer RNA-seq expression matrix data, and pan-cancer clinical information were downloaded from http://github.com/greenelab/pancancer (accessed on 16 March 2023). The data cleaning and screening processes were carried out according to the methods described in a previous study [73]. A total of 18 of 33 cancer types with a higher fraction of the TP53 mutation were employed in this study. The neural network prediction model uses the 97 intersection genes of the top 100 specific genes in SFRP2^+^ fibroblast and TCGA expression profile data as the input layer. The neural network contained three hidden layers and one output layer. The first hidden layer contains 512 neurons, the second hidden layer contained 256 neurons, and the third hidden layer contained 128 neurons, using the ReLU activation function. The ReLU activation function was used in each of the hidden layers. The output layer, which contains 2 neurons, used the Softmax activation function to predict whether there is a *TP53* mutation in tumor patients. The neural network model using a train-to-test dataset ratio of 4:1, Cross-Entropy as a loss function, and RMSprop as an optimizer was trained for 50 epochs.

## Data Availability

R and python codes are available from the corresponding author. All original data are available in GEO and TCGA database.
